# Genetic alterations in myeloid sarcoma among acute myeloid leukemia patients: insights from 37 cohort studies and a meta-analysis

**DOI:** 10.3389/fonc.2024.1325431

**Published:** 2024-03-01

**Authors:** Suvijak Untaaveesup, Sasinipa Trithiphen, Kamolchanok Kulchutisin, Tarinee Rungjirajittranon, Nattawut Leelakanok, Sujitra Panyoy, Thanapon Kaokunakorn, Weerapat Owattanapanich

**Affiliations:** ^1^ Paholpolpayuhasena Hospital, Department of Medical Organization, Kanchanaburi, Thailand; ^2^ Division of Hematology, Department of Medicine, National Cancer Institute Thailand, Bangkok, Thailand; ^3^ Faculty of Medicine Siriraj Hospital, Mahidol University, Bangkok, Thailand; ^4^ Division of Hematology, Department of Medicine, Faculty of Medicine Siriraj Hospital, Mahidol University, Bangkok, Thailand; ^5^ Center of Excellence of Siriraj Adult Acute Myeloid/Lymphoblastic Leukemia, Faculty of Medicine Siriraj Hospital, Mahidol University, Bangkok, Thailand; ^6^ Division of Clinical Pharmacy, Faculty of Pharmaceutical Sciences, Burapha University, Chonburi, Thailand; ^7^ Department of Medicine, Chao Phraya Yommaraj Hospital, Suphanburi, Thailand

**Keywords:** acute myeloid leukemia, extramedullary blast, genetic, mutation, myeloid sarcoma

## Abstract

**Introduction:**

Variations in mutation rates among acute myeloid leukemia (AML) patients with myeloid sarcoma (MS) underscore the need for a thorough examination. This meta-analysis was conducted to fill the information gap concerning mutation frequencies in AML patients presenting with MS.

**Materials and methods:**

This study included retrospective and prospective cohorts. It examined genetic alterations in AML patients with and without MS across all age groups. The search strategy employed terms such as “acute myeloid leukemia,” “extramedullary,” “granulocytic sarcoma,” “myeloid sarcoma,” and “leukemic cutis” in the EMBASE, MEDLINE, and Scopus databases. Excluded from the study were reviews, case reports, and case series with fewer than 10 cases. Statistical analyses were performed with Review Manager 5.4 software.

**Results:**

The primary analysis incorporated data from 37 cohorts involving 5646 diagnosed AML patients and revealed a 17.42% incidence of MS. The most prevalent mutation among AML patients with MS was *FLT3*-ITD, with a pooled prevalence of 17.50% (95% CI 12.60% to 22.50%; I^2^ 82.48%). The dominant fusion gene was *RUNX1::RUNX1T1*, displaying a pooled prevalence of 28.10% (95% CI 15.10% to 41.20%; I^2^ 96.39%). In comparison, no significant intergroup differences were observed for *NPM1*, *FLT3*-ITD, *KIT*, and *IDH2* mutations. Interestingly, the *CEBPA* mutation exhibited protective effects for MS patients, with an odds ratio of 0.51 (95% CI 0.32 to 0.81; I^2^ 0%). Conversely, the *NRAS* mutation was associated with an increased risk of MS development, with an odds ratio of 5.07 (95% CI 1.87 to 13.73; I^2^ 0%).

**Conclusion:**

This meta-analysis sheds light on the prevalence of genetic mutations in AML patients with MS, providing insights into the unique characteristics of the mutations and their frequencies. These discoveries are crucial in informing therapeutic and prognostic decisions for individuals with myeloid sarcoma.

## Highlights

- Data from 37 cohorts, consisting of 6475 acute myeloid leukemia (AML) patients, were analyzed to determine the genetic profile of AML patients with myeloid sarcoma (MS).
- FLT3-ITD is the most prevalent mutation, and RUNX1::RUNX1T1 is the most common fusion gene in AML patients with MS.
- The CEBPA mutation offers protective effects to MS patients, while the NRAS mutation heightens the risk of MS development.

## Introduction

Acute myeloid leukemia (AML) is characterized by the uncontrolled proliferation of myeloid stem cells and impaired differentiation (1). In 2019, the United States observed an estimated total of more than 20 000 AML cases, with certain studies suggesting an age-adjusted incidence rate of 3.43 cases per 100 000 individuals annually (2). Extensive research into AML pathogenesis has identified numerous mutations and cytogenetic abnormalities as pivotal contributors to disease onset (3, 4). In 2016, the World Health Organization classified myeloid sarcoma (MS) as an AML subtype. This classification was retained in the World Health Organization’s updated 2022 classification and the 2022 International Consensus Classification, wherein MS remains a recognized entity (5–7).

MS is a tumor mass formed of myeloblasts outside the bone marrow (3). Predominantly, MS affects patients diagnosed with AML or chronic myeloid leukemia, constituting approximately 9% of these cases (8). Notably, the prevalence of MS is greater in males than females, and the condition predominantly affects individuals aged 46 to 59 years (3, 8, 9). The pathophysiology of MS, especially the migration of cells to extramedullary sites, remains elusive. Prevailing hypotheses suggest that the development of MS may be linked to leukemic cells expressing CD56 (neural cell adhesion molecule) (4). These cells possibly bind to tissues commonly associated with MS manifestations (3).

MS commonly manifests in extramedullary sites such as the skin, bones, soft tissues, and gall bladder (4). However, some studies also document its occurrence in rarer locations, including the pleura, penis, and vulva (10–12). The prognosis for MS patients tends to be unfavorable and can vary based on the location of the lesion and its molecular attributes (1, 8, 13). In modern diagnostic methodologies, next-generation sequencing (NGS) has emerged as a crucial tool for identifying mutations in AML patients, including those with MS (3). The *NPM1* mutation is the most common mutation found in MS; other common mutations and fusion genes include *KRAS*, *NRAS*, *KIT*, *CEBPA*, *IDH1*, *IDH2*, *RUNX1::RUNX1T1*, and *CBFB::MYH11* (3, 10, 14–16). However, variations persist in the reported incidence of each mutation in MS among studies (12, 14, 16). Moreover, a previous report indicated variations in the prevalence of chromosomal abnormalities and/or molecular mutations among different countries (17). However, there is currently no available data regarding these variations specifically within the subgroup of AML with MS.

Consequently, this systematic review and meta-analysis compiled and analyzed data on the incidence of each mutation from all pertinent sources. Our objective was to better understand the specific characteristics and precise prevalence of genetic mutations in AML patients presenting with MS.

## Materials and methods

### Data sources and searches

Six researchers (S.U., K.K., S.P., T.K., W.O., and T.R.) independently searched for articles published within the EMBASE, MEDLINE, and Scopus databases from their inception up to August 1, 2023. The search terms included “acute myeloid leukemia,” “extramedullary,” “granulocytic sarcoma,” “myeloid sarcoma,” and “leukemic cutis.” A comprehensive description of the search strategy is provided in **Supplementary Data 1**. Our systematic review and meta-analysis strictly followed the PRISMA (Preferred Reporting Items for Systematic Reviews and Meta-Analyses) guidelines, as elaborated in **Supplementary Data 2**.

### Selection criteria and data extraction

Studies included in the meta-analysis were retrospective or prospective cohort studies on AML with MS and with a primary outcome aligned with our research objective. We excluded reviews, case reports, and case series with fewer than 10 cases. The primary objective of this analysis was to determine the incidence of each mutation in AML patients with MS, while the secondary aim was to compare the mutational statuses of AML patients with and without MS. To ascertain study eligibility, four researchers (S.U., K.K., S.P., and T.K.) independently assessed the titles and abstracts of the retrieved studies. They also reviewed the references in the selected studies to identify any additional pertinent research. In instances of disagreement about the inclusion of specific studies, consensus was reached through mediation with two other investigators (W.O. and T.R.).

### Quality assessment

The quality assessment of the included studies was independently conducted by two researchers (S.U. and K.K.) using the Newcastle–Ottawa quality assessment scale (18).

### Statistical analysis

We analyzed the data with Review Manager 5.4 software provided by the Cochrane Collaboration (London, United Kingdom). The inverse variance method was employed to compute pooled odds ratios (ORs) and their corresponding 95% confidence intervals (CIs) for each gene across the studies (19). The prevalence of the genetic alterations was meta-analyzed and pooled using the binary random-effects model using the DerSimonian–Laird method (Open Meta–Analyst for Windows 8) (20). Given the anticipated variability among the incorporated studies, a random-effects model was favored over a fixed-effects model for our meta-analysis. We evaluated statistical heterogeneity with Cochran’s Q test and quantified its extent using the I^2^ statistic. Depending on the I^2^ values, heterogeneity was classified as either insignificant (0%–25%), low (25%–50%), moderate (50%–75%), or high (75%–100%) (19). For transparency and procedural clarity, we registered our study protocol with the International Platform of Registered Systematic Review and Meta-Analysis Protocols (INPLASY) network (registration number INPLASY202380091).

## Results

### Search results

**Figure 1:**
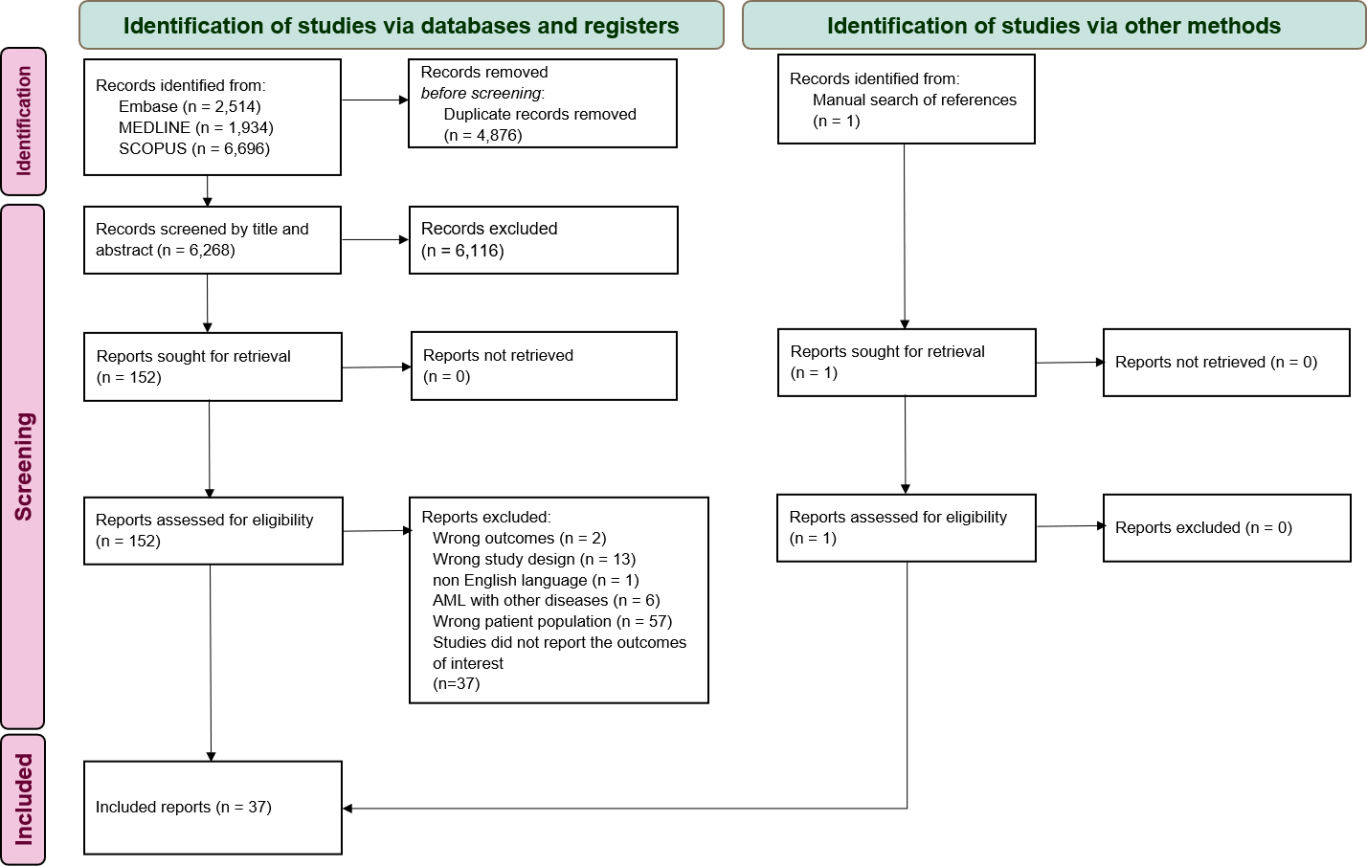
Flowchart depicting the literature review and article selection process.

A total of 11 145 articles were identified in the search process, with 1934 articles from MEDLINE, 2514 from EMBASE, 6696 from Scopus, and 1 from other sources. Initially, 4876 duplicated articles were removed, and another 6269 were excluded after reviewing the titles and abstracts. The remaining 153 articles underwent a thorough full-text reading. This resulted in a further 116 articles being excluded because they did not meet the inclusion criteria. The remaining 37 articles that met the inclusion criteria were included in our analysis. The data gathering and screening process is depicted in **Figure 1**.

### Baseline patient characteristics

The analysis included 37 cohorts that collectively comprised 6475 diagnosed AML patients. Among these patients, 907 out of 5206 (17.42%) were identified as having AML with MS, resulting in 2199 MS cases. **Table 1** details the characteristics and quality assessments of the analyzed cohorts. There was a slight male predominance, accounting for approximately 58.26%, with females comprising 41.74%. When analyzing age demographics across cohorts, 71.87% of the patient population was over 18 years old, while the remaining 28.13% was under 18. Further analysis indicated that the most common sites of MS manifestation were the skin, orbit, central nervous system, and lymph nodes.

**Table 1:** Summary of baseline patient characteristics of each included article.

References	Geographical distribution	Numbers (case/control)	Sex (M/F)	Median age (range)	Techniques/List genes in the study	Locations of extramedullary AML of cases (n)	Treatment (n)	Stem cell transplant (n)	Study period	Type	Newcastle-Ottawa scale
**Ansari-Lari 2004** (21)	USA	20/-	13/7	41yr.(4mo.-84yr.)	PCR/ *FLT3*-ITD, *FLT3*-D835	skin(8), lymph node(5), breast(2), nasal cavity(1),brain(1), ileum(1), pericardium(1), testis(1), retroperitoneum(1)	NA	NA	NA	R	Selection:3 Comparability: 0 Outcome:3
**Creutzig 2012** (22)	Germany, Austria,Switzerland	137/-	NA	(<18)	NA/ *CBF, MLL* rearrangement	Skin(39), orbit(36), kidney (42), tonsil(16),Salivary gland(6), testis(7), multiple site(55)	induction high dose cytarabine (137)	NA	1998-2010	R	Selection:3 Comparability: 0 Outcome:3
**Ohanian 2012** (23)	USA	10/-	6/4	49(19-79)	Cytogenetics, FISH, PCR/ *NPM1, NRAS*	orbit and ocular adnexae(10), CSF(1), breast(1),skin(3), lymph node(1), lung/mediastinum(2),bone(1)	IC (9),RT(2),surgery(5)	allo-SCT (3)	NA	R	Selection:3 Comparability: 0 Outcome:3
**Pemmaraju 2012** (16)	USA	244/-	135/109	57(14-82)	Cytogenetics, PCR/ *FLT3, NRAS, KRAS, NPM1, CBFb::MYH11, CEBPA, JAK2, IDH1, IDH2, KIT*	skin(84),CNS(78),RS(43),GI(23),lymph node(21), soft/connective tissue(27), pelvis/inguinal(15),naso-oropharynx(8),musculoskeletal(7),CVS(4),urinary(4),eye(1),thymus(1)	NA	NA	2000-2011	R	Selection:3 Comparability: 0 Outcome:3
**Tran 2012** (24)	USA	9/-	3/6	45 (28-69)	Cytogenetics, PCR/ *FLT3, NPM1, JAK2, RAS, CBFB-MYH11*	ovary(2),uterus(2),fallopian tube (2), ureter(1),parametrial soft tissue(1), breast (1),lymph node(1),bladder (1),epididymis(1),labia(2), pleural fluid (1), kidney(2),testicle(1),spermatic cord(1), nasopharynx(1), skin(1), chest soft tissue(1)	NA	NA	2000-2011	R	Selection:3 Comparability: 0 Outcome:3
**Gupta 2013** (25)	India	9/-	6/3	(9mo.-18yr)	Cytogenetics/t(8;21)	orbit(4), maxilla,mandible, porta-hepatis,urinary bladder,spinal cord (1),paravertebral muscles(1), pre-sternal region(1), retro-sternal region(1), uterus(1), craniofacial sinuses(1)	NA	NA	2006-2012	R	Selection:3 Comparability: 0 Outcome:2
**Wang 2013** (26)	USA	63/-	34/29	50 (1-80)	PCR/ *FLT3, NRAS, KRAS, KIT, NPM1, CEBPA, JAK, IDH1, IDH2*	lymph node,skin	IC (63)	11	2002-2012	R	Selection:3 Comparability: 0 Outcome:3
**Luskin 2015** (27)	USA	75/769	160/124	59(17-86)	NGS, PCR/ 33 gene panel list	skin(27)	NA	NA	2001-2014	R	Selection:4 Comparability: 1 Outcome:3
**Goldberg 2017** (28)	USA	17/-	10/7	56 (26-86)	NGS/ 585 list genes	soft tissue(4),lymph node(3),bladder(1),GI(3),breast(2),testis(1),gingiva(1),fallopian tube(1),paratracheal/neck(1)	NA	NA	NA	P	Selection:3 Comparability: 0 Outcome:1
**Støve 2017** (29)	Denmark, Finland, Iceland, Norway, Sweden and Hong Kong	73/-	36/37	2.6 (0.1-17.9)	Cytogenetics, PCR/ *FLT3-*ITD*, FLT3-*ALM*, FLT3*-wild type*, NPM1, NPM1-*wild type*, CBFB::MYH11, RUNX1::RUNX1T1, MLLT3::KMT2A*	skin(16), orbita(11), lymph nodes(5), gingiva/mouth(3), abdomen(2), dura/epidural space(2), sinus(1), mandible(1), maxilla(1), the mastoid process(1), neck(1), humerus(1), mediastinum(1),lung(1), pericardium(1), pancreas(1), appendix(1), retroperitoneum(1), kidney(1), bilateral adrenal glands(1), labia majora(1), bilateral testes(1),gluteal region(1), thigh(1)	IC (73)	SCT (13)	2004-2013	R	Selection:4 Comparability: 2 Outcome:3
**Wu 2017** (30)	China	18/-	NA	(8-61)	PCR/ *FLT3-*ITD*, RUNX1::RUNX1T1*	NA	NA	NA	NA	R	Selection:3 Comparability: 0 Outcome:3
**Choi 2018** (31)	Korea	13/-	5/8	46 (18-83)	NGS/ 83 gene panel list	skin(2), lymph node(4), breast(2), nasopharynx (1),leptomeningeal(1), Right ventricle(1), axilla(2),frank(1), inguinal area(1), stomach(1), right frontal lobe(1),scalp(1),ovary(1), bone(5),mesentery(1), anterior chest(4), paravertebral(1), intramuscular nodule(1), lung(1), gingiva(1), scortum(1)	NA	NA	2003-2016	R	Selection:3 Comparability: 0 Outcome:1
**Claerhout 2018** (14)	Belgium	41/-	23/18	48(0.8-86)	PCR/ *FLT3-*ITD*, JAK2 V617F, RUNX1::RUNX1T1, CBFB::MYH11, KMT2A::MLLT3*	skin & subcutaneous tissue(14),lymph node(10), GItract(6),eye/orbita(3), breast(4),mediastinum(4), retroperitoneum(1),ovary(2),lung(1),cervix/uterus(1), spinal cord(1),urinary tract(1),pericard(1),brain(2), thyroid(1),liver(1),bone(2)	IC (10), IC then allo SCT(10),other regimen (20)	AlloSCT(10)	1983-2016	R	Selection:3 Comparability: 0 Outcome:3
**Kaur 2018** (11)	USA	23/-	16/7	58 (36-84)	FISH, NGS/*FLT3, ASXL1, STAG2, JAK2, TP53*	skin(12), scalp(2), lymph node, chest wall(1), vulva(1), penis(1), axilla(2), gum(1), spleen(1), small intestine, humerus(1), abdomen(1),leg(1)	IC (19),splenectomy(1),NA(3)	alloHSCT(5)	2002-2015	R	Selection:3 Comparability: 0 Outcome:3
**Lee 2018** (32)	Taiwan	25/-	14/11	45(17-72)	NGS/ 54 gene panel list	NA	CMT (20)	alloSCT(9)	2005-2018	R	Selection:3 Comparability: 0 Outcome:3
**Pramanik 2018** (9)	India	121/449	NA	6 (0.3-18)	Cytogenetics, PCR/ *FLT3, NPM1, RUNX1::RUNX1T1, CBFB::MYH11, MLL* rearrangement*, DEK::NUP, BCR::ABL*	orbit(107), CNS(5), skin(1), lung/pleura(1), jaw(1), mediastinum(1), testis(1), ear(1), lung(1)	CMT(121)	NA	2003-2016	R	Selection:4 Comparability: 2 Outcome:3
**Wang 2019** (33)	USA	62/186	33/29	58.2	Cytogenetic, PCR/ *NPM1, FLT3-*ITD*,MLL* rearrangement	skin(62)	NA	NA	2005-2017	R	Selection:4 Comparability: 2 Outcome:3
**Andrew 2020** (34)	Canada	158/377	98/60	57.58(19-89)	Cytogenetics, PCR/ *FLT3*-ITD*, NPM1, RUNX1::RUNX1T1*	skin(57),lymphatic system(36),abdomen(19),CNS(15),reproductive system(8),lung(6)	IC (125)	NA	2000-2019	R	Selection:4 Comparability: 2 Outcome:3
**Hu 2020** (35)	China	44/170	33/11	NA	Cytogenetics, PCR/ *NPM1, CEBPA, GATA1, c-KIT, RUNX1::RUNX1T1, CBFB::MYH11*	orbit(22), CNS(15), bone(8), skin(7), lymph nodes(4), mediastinum(3), lung/pleura(2), abdominal cavity(2)	IC	NA	2008-2018	R	Selection:4 Comparability: 2 Outcome:3
**Karagounis 2020** (36)	USA	11/-	NA	66(26-82)	NGS, PCR/ 44 list gene panel, *FLT3*	skin(11)	NA	NA	2007-2017	R	Selection:3 Comparability: 0 Outcome:3
**Xu 2020** (37)	NA	109/775	60/49	5.8(<1-18)	Cytogenetics, PCR/ *FLT3*-ITD*, CEBPA, NPM1*	CNS(15)	IC (109)	SCT(16)	1996-2010	R	Selection:4 Comparability: 2 Outcome:3
**Zhou 2020** (38)	USA	33/-	24/9	2.8yr.(1mo.- 18yr.)	NGS/ 152 gene panel list	skin(18),soft tissue(9), head andneck(6),extremities(3),bone(3),lymph node(2),orbit(2),breast(1),lung(1),bladder(1),testis(1),lacrimal gland(1),CNS (brain/spine)(1)	NA	NA	1984-2016	R	Selection:3 Comparability: 0 Outcome:3
**Abbas 2021** (39)	USA	56/-	36/20	58(21-79)	NGS, FISH/ *NRAS, KRAS, DNMT3A, ASXL1, NPM1, CEBPA, IDH2, JAK2, PTPN11, TET2, BCOR, RAD21, FLT3, EZH2, TP53, KMT2A, RUNX1*	skin(19),musculoskeletal(13), lymph node(12),GI(8), GU(8),breast(3),head and neck(6),other(3)	IC (39), surgery(1), low intensive treatment (10), no treatment (3),venetoclax based regimen+/-RT(10), CMT+RT(5)	AlloSCT 10/53	2005-2020	R	Selection:3 Comparability:0 Outcome:3
**De Cap 2021** (40)	USA	96/-	60/36	63(20-86)	NA/ *NPM1, RUNX1, ETV6, FLT3, NRAS, JAK2, DNMT3A, TET2, IDH1, IDH2, ASXL1, SRSF2, U2AF1*	Skin&oropharyngeal mucosa(45), lymph node(17),bone &soft tissue(30),other(29)	NA	NA	NA	R	Selection:3 Comparability: 0 Outcome:3
**Goyal 2021** (41)	India	28/-	18/10	22(1.8-76)	Cytogenetics, PCR/ *NPM1, RUNX1::RUNX1T1*	lymphatic system(8), CNS(7), GIT(5), bone&soft tissue(3), skin(2), multiple sites(3)	IC (26)	NA	2012-2021	R	Selection:3 Comparability: 0 Outcome:3
**Greenland 2021** (15)	USA	7/-	4/3	48(19-84)	NGS/ *KMT2A, SETD2, ASXL1, STAG2, SMC3, IDH2, TET2, FLT3, NRAS, BRAF, SRSF2, CEBPA, BCORL1, BCOR, CUX1, TP53, WT1, NF1, NPM1*	kidney(2),lung(1),liver(1), small intestine(1), cutaneous(2),bone(1), testicle(1),lymph nodes(1),periaortictissue(1),gallbladder(1)	Allograft transplant(5),CMT (1)	5	2007-2017	R	Selection:3 Comparability:0 Outcome:3
**Halahleh 2021** (42)	Jordan	32/-	22/10	33.5(1-63)	NGS, PCR/ 52 list gene panel, *FLT3*	NA	IC (29), Surgical resection(2),RT(6)	16	2003-2019	R	Selection:3 Comparability: 0 Outcome:3
**Khan 2021** (43)	USA	10/-	7/3	1-79	NGS/*KMT2A::MLL, ASXL1, TET2, NRAS, CEPBA, TP53, MLLT1::ENL, MLLT3, MLLT10::AF10, ASXL1, CEBPA, PHF6, BRCA2, DNMT3A*, *NPM1, RAD21, CBL, KMD6A, NF1*	skin(7), soft tissue(3)	NA	NA	2014-2021	R	Selection:3 Comparability: 0 Outcome:1
**Tatarian 2021** (44)	USA	25/23	NA	NA	NGS/ *FLT3*	CNS(25)	intrathecal CMT (10)	NA	2015-2020	R	Selection:3 Comparability: 0 Outcome:1
**Velagala 2021** (45)	India	44/-	29/15	95mo.(32mo.-178mo.)	FISH, PCR/*RUNX1::RUNXT1, CBFB::MYH11, KMT2A-r, FLT3-*ITD	orbital(27), para-spinal(6)	IC (44),RT (25)	NA	2014-2019	R	Selection:2 Comparability: 0 Outcome:3
**Eckardt 2022** (10)	NA	225/1358	119/106	53(42-61)	NGS, PCR/ *NPM1, FLT3-*ITD*, PTPN11, IDH2, CEBPA, RUNX1::RUNX1T1, CBFB::MYH11*	CNS(10),tonsils(2),pleura(5),liver(2),testes(1),skin(17),spleen(1),pericardium(2),lymph nodes(3)	allogeneic hct(66)	allogeneic hct(66)	NA	R	Selection:4 Comparability: 2 Outcome:3
**Kim 2022** (46)	Korea	35/86	23/12	7.87	Cytogenetics, PCR/ *C-kit, FLT3-*ITD*, NPM1, CEBPA, CBFB::MYH11, MLL, RUNX1::RUNX1T1*	Head and neck(22),trunk (12), musculoskeletal(35)	NA	NA	2009-2018	R	Selection:4 Comparability: 2 Outcome:3
**Zhao 2022** (12)	China	118/-	73/45	44(1-81)	NGS/ 18 gene panel list	lymph nodes(30), soft tissues(16),spinal canal(14),digestive tract(9), genitalsystem(8),pleura(7),skin(2),nasopharynx(7),lung(5),bone(3),brain(1),breast(3),mediastinum(3),orbit(3),gingiva(3),parotid(3),other(2)	local treatment(30), CMT (60)	allo-SCT(9)	2010-2021	R	Selection:3 Comparability: 0 Outcome:3
**Kuhlman 2022** (47)	USA	83/-	52/31	56(17-89)	NGS/ *RTK-RAS, NPM1, TET2, IDH2*	NA	Induction CMT (70), IC+alloSCT, IC+LT	NA	1996-2021	R	Selection:3 Comparability: 0 Outcome:3
**Ye 2022** (48)	China	11/-	8/3	7	FISH, PCR, NGS/ NA	Skin(3), orbital(3), LN(2), CNS(3), testis(1), mediastinum(1)	Sx(9), CMT(10), HSCT(3), TKI(2)	3	2016-2022	R	Selection: 4 Comparability: 0 Outcome: 3
**Yang 2023** (49)	China	61/-	36/25	37(8-87)	NGS, PCR/ *C-kit, NPM1, ETV6, TET2*, *IDH2, RUNX1, CEBPA, FLT3-*ITD, *TP53, KRAS, CALR, RUNX1::RUNX1T1, CBFB::MYH11, BCR::ABL, ETV6::MECOM, FUS::ERG, PICALM-MLLT10*	LN(19), Soft tissue(15), Bone and joints(9), Mediastinum(7), CNS(6), orbit(4), pleural and abdominal cavity(4), ovaries(3), Breast(3), oral cavity(2), sinus(2), testis(2), GI(2), kidney(2), liver(1)	CMT(47), Sx(11), Targeted therapy(7), Allo SCT(10), RT(8)	10	2015-2020	R+P	Selection: 3 Comparability: 0 Outcome: 3
**Owattanapanich 2023** (50)	Thailand	53/106	25/28	54.3±15.5	NGS, PCR/ *ABL1, ANKRD26, ASXL1, CALR, CBL*, *CEBPA, CSF3R, DDX41, DNMT3A, EZH2*, *FLT3, GATA2, IDH1, IDH2, JAK2, KIT, KRAS*, *MECOM, MPL, NPM1, NRAS, PTPN11* *(RPL6), RUNX1, SAMD9, SAMD9L SETBP1*, *SF3B1, SH2B3, SRSF2, TET2, TP53, U2AF1*, *WT1,ZRSR2*	Skin(53), Spleen(12), LN(10), Liver(9)	IC (104),HMA(13), transfusion support, HU, cytarabine	NA	2013-2020	R	Selection: 3 Comparability: 1 Outcome: 3

allo-SCT, allogeneic stem cell transplant; AML, acute myeloid leukemia; CMT, chemotherapy; F, female; FISH, fluorescence *in situ* hybridization; HSCT, hematopoietic stem cell transplantation; IC, intensive chemotherapy; LT, localized therapy; M, male; NA, not applicable; NGS, next-generation sequencing; P, prospectively; PCR, polymerase chain reaction; R, retrospectively; SCT, stem cell transplant; TKI, tyrosine kinase inhibitors.

### Pool prevalence of DNA mutations in AML patients with MS

**Figure 2:**
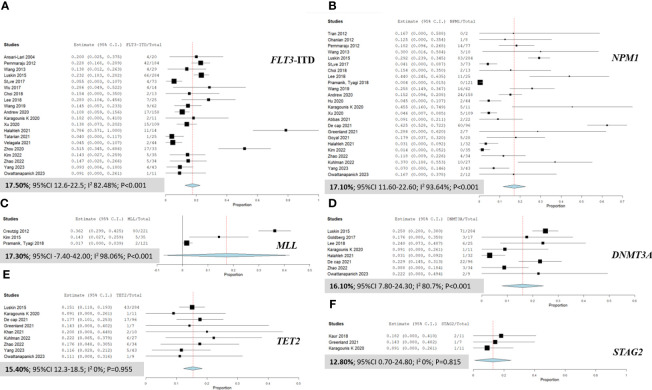
Forest plots illustrating the prevalence of gene mutations in AML patients with myeloid sarcoma. **(A)** *FLT3*-ITD; **(B)** *NPM1*; **(C)** *MLL*; **(D)** *DNMT3A*; **(E)** *TET2*; and **(F)** *STAG2*.

Our detailed analysis of the included articles provided comprehensive insights into the pooled prevalence of molecular mutations among patients diagnosed with MS. **Figure 2** presents the significant molecular mutations extracted from this extensive dataset. Among them, the *FLT3*-ITD mutation was the most prevalent, with a pooled prevalence of 17.50% (95% CI 12.60% to 22.50%; I² 82.48%; **Figure 2A**) (12, 16, 21, 26, 27, 29–34, 36–38, 42, 44–46, 48, 50). Similarly, the *MLL* and *NPM1* mutations stood out with a prevalence of 17.30% (95% CI -7.40% to 42.0%; I² 98.06%; **Figure 2C**) (9, 22, 46) and 17.10% (95% CI 11.60% to 22.60%; I² 93.64%; **Figure 2B**) (9, 12, 15, 16, 23, 24, 26, 27, 29, 31–37, 39–42, 46), respectively. Additionally, the *DNMT3A* mutation was observed at a prevalence of 16.10% (95% CI 7.80% to 24.30%; I² 80.7%; **Figure 2D**) (12, 27, 28, 32, 36, 40, 42, 50), while the *TET2* mutation had a prevalence of 15.40% (95% CI 12.30% to 18.50%; I² 0%; **Figure 2E**) (12, 15, 27, 36, 40, 43, 47, 49, 50). Furthermore, the *STAG2* and *NRAS* mutations exhibited a prevalence of 12.80% (95% CI 0.70% to 24.80%; I² 0%; **Figure 2F**) (11, 15, 36) and 11.9% (95% CI 8.10% to 15.70%; I² 39.18%), respectively. For a more detailed exploration of the pooled prevalence of DNA mutations in MS, please refer to **Table 2**.

**Table 2:** Pooled prevalence of gene mutations in AML patients with myeloid sarcoma.

Molecular mutations	Number of included studies	% (95% CI)	I^2^
*NPM1*	24	17.10 (11.60-22.60)	93.64
Signal transduction pathway			
*FLT3*-ITD	19	17.50 (12.60-22.50)	82.48
*NRAS*	12	11.90 (8.10-15.70)	39.18
*KIT*	9	9.90 (4.90-15.00)	75.60
*FLT3*-TKD	9	6.60 (3.60-9.60)	37.04
*PTPN11*	2	6.40 (0.90-12.00)	64.79
*JAK2*	8	4.70 (2.20-7.20)	8.84
*KRAS*	6	3.80 (2.20-5.40)	0
*SH2B3*	2	3.30 (-2.00-8.70)	0
*CBL*	4	2.20 (0.70-3.70)	0
*BRAF*	2	1.00 (-4.90-7.00)	9.84
Myeloid transcription factor			
*BCORL1*	2	10.70 (-3.60-24.90)	0
*RUNX1*	7	6.60 (2.20-11.00)	58.38
*ETV6*	4	5.30 (0.80-9.70)	58.06
*CEBPA*	10	3.30 (0.90-5.80)	18.41
Tumor suppressor gene			
*WT1*	3	5.90 (3.20-8.50)	0
*TP53*	9	4.90 (2.00-7.70)	48.78
*NF1*	2	3.60 (-2.10-9.30)	0
*PHF6*	2	1.50 (0.20-2.80)	0
Epigenetic modifier			
*MLL*	3	17.30 (-7.40-42.0)	98.06
*DNMT3A*	8	16.10 (7.80-24.30)	80.70
*TET2*	9	15.40 (12.30-18.50)	0
*KMT2A*	3	8.40 (-0.30-17.00)	46.22
*IDH2*	9	9.80 (5.00-14.50)	61.24
*ASXL1*	10	7.30 (5.10-9.50)	0
*IDH1*	8	5.70 (2.30-9.10)	57.15
*EZH2*	3	4.60 (-2.80-12.00)	62.33
*SETD2*	2	3.60 (-2.10-9.30)	0
*SETBP1*	2	3.30 (-2.00-8.50)	0
Spliceosome gene			
*SRSF2*	5	7.10 (3.00-11.30)	4.39
*U2AF1*	4	4.70 (1.30-8.00)	0
*SF3B1*	2	4.20 (1.90-6.60)	0
Cohesion gene			
*STAG2*	3	12.80 (0.70-24.80)	0

### Pool prevalence of fusion genes in AML patients with MS

**Figure 3:**
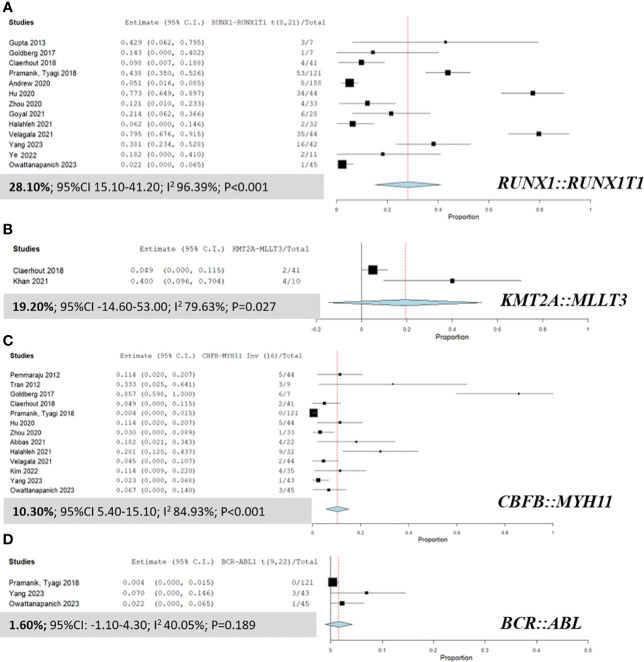
Forest plots displaying the prevalence of fusion genes in AML patients with myeloid sarcoma. **(A)** *RUNX1::RUNX1T1*; **(B)** *KMT2A::MLLT3*; **(C)** *CBFB::MYH11*; and **(D)** *BCR::ABL*.

**Figure 3** presents a detailed analysis of the pooled prevalence of fusion genes in patients with MS, highlighting the frequency of various fusion genes within this group. The most predominant fusion gene observed was *RUNX1::RUNX1T1*, with a remarkable pooled prevalence of 28.10% (95% CI 15.10% to 41.20%; I² 96.39%; **Figure 3A**) (9, 14, 25, 28, 34, 35, 38, 41, 42, 45, 48–50). The *KMT2A::MLLT3* fusion gene was also identified at a pooled prevalence of 19.20% (95% CI -14.60% to 53.00%; I² 79.63%; **Figure 3B**) (14, 43). Furthermore, the *CBFB::MYH11* fusion gene was observed at a pooled prevalence of 10.30% (95% CI 5.40% to 15.10%; I² 84.93%; **Figure 3C**) (9, 14, 16, 24, 28, 35, 38, 39, 42, 45, 46, 49, 50).

### Subgroup analysis

When categorizing patients into two age groups (<40 years and ≥40 years), we observed distinct patterns of mutations. Among patients under 40 years of age, *KRAS* mutation emerged as the most prevalent, occurring in 50.00% of cases (95% CI 10.00% to 90.00%; I² 0%). Conversely, in individuals aged 40 years and above, *SRSF2* mutation was the most commonly observed, with a pooled prevalence of 21.90% (95% CI -0.30% to 44.20%; I² 0%). **Supplementary Data 3** provides a detailed breakdown of the prevalence of molecular mutations and fusion genes within these age groups.

Furthermore, we calculated the overall pooled prevalence of AML patients with MS harboring recurrent genetic abnormalities, as per the 2022 World Health Organization classification, to be 36.80% (95% CI: 26.00% to 47.60%; I² 96.85%; **Supplementary Data 4**) (9, 12, 14–16, 23–29, 31–43, 45–50).

The prevalence of genetic mutations, stratified by geographical distribution, was investigated. Among Western patients, the third most frequently observed mutations were *NPM1* (27.50%; 95% CI: 17.80 to 37.30; I² 87.18%), *FLT3*-ITD (20.50%; 95% CI: 13.90 to 27.10; I² 77.52%), and *KMT2A* (19.90%; 95% CI: -15.00 to 54.90; I² 72.89%). Conversely, in Eastern patients, the most common mutations were *KRAS* (20.10%; 95% CI: 8.90 to 31.30; I² 7.93%), *FLT3*-ITD (18.10%; 95% CI: 5.90 to 27.20; and I² 82.73%), and *KIT* (15.20%; 95% CI: 9.40 to 21.00; I² 0%) mutations. *CBFB::MYH11* emerged as the predominant fusion gene in the Western population, while *RUNX1::RUNX1T1* predominated in the Eastern population, with rates of 20.40% (95% CI: 7.20 to 33.50; I² 88.31%) and 21.50% (95% CI: 10.10 to 32.90; I² 95.69%), respectively. The genetic profiling of AML patients with MS in both Western and Eastern countries is presented in **Supplementary Data 5,** **6**.

### Comparison of mutational profiles between the AML with and without MS groups

**Figure 4:**
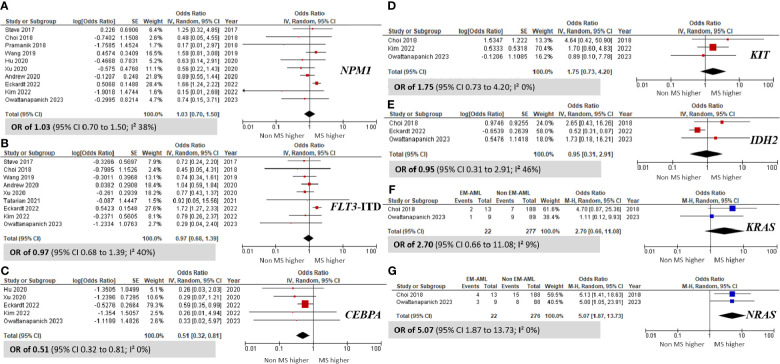
Forest plots indicating the prevalence of gene mutations in AML patients with myeloid sarcoma in comparison to those without myeloid sarcoma. **(A)** *NPM1*; **(B)** *FLT3*-ITD; **(C)** *CEBPA*; **(D)** *KIT*; **(E)** *IDH2*; **(F)** *KRAS*; and **(G)** *NRAS*.

Several noteworthy findings emerged after analyzing gene mutations in patients with MS and non-MS (**Figure 4**). Specifically, the prevalence of the *CEBPA* mutation was significantly higher in non-MS patients than in those with MS, with an OR of 0.51 (95% CI 0.32 to 0.81; I² 0%; **Figure 4C**) (10, 35, 37, 46, 50). Conversely, the *NRAS* mutation was notably more prevalent in the MS group, with an OR of 5.07 (95% CI 1.87 to 13.73; I² 0%; **Figure 4G**) (31, 50). However, no significant differences were observed in the prevalence of the *NPM1*, *FLT3*-ITD, *KIT*, and *IDH2* mutations between MS and non-MS patients.

**Figure 5:**
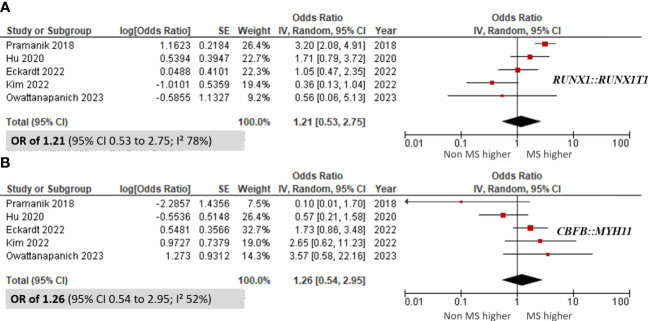
Forest plots showing the prevalence of fusion genes in AML patients with myeloid sarcoma in comparison to those without myeloid sarcoma. **(A)** *RUNX1::RUNX1T1*; and **(B)** *CBFB::MYH11*.

Additionally, we assessed the incidence of the *RUNX1::RUNX1T1* and *CBFB::MYH11* fusion genes in four included studies. The meta-analysis revealed no significant differences in the incidence of these fusion genes between patients with MS and those without MS, with pooled ORs of 1.21 (95% CI 0.53 to 2.75; I² 78%; **Figure 5A**) (9, 10, 35, 46, 50) for *RUNX1::RUNX1T1* and 1.26 (95% CI 0.54 to 2.95; I² 52%; **Figure 5B**) (9, 10, 35, 46, 50) for *CBFB::MYH11*. Furthermore, there was no significant correlation between AML patients harboring MS and the presence of recurrent genetic abnormalities (pooled OR 0.74; 95% CI 0.42 to 1.29; I² 82%; **Supplementary Data 4**) (9, 29, 31, 33, 34, 37, 46, 50).

## Discussion

MS, commonly known as extramedullary AML, presents a wide range of clinical manifestations and often poses therapeutic challenges. A prior multicenter cohort study documented an MS incidence of 14.21% among newly diagnosed AML cases. Typically, pivotal therapeutic choices are guided by the genetic alteration profile. This current study marks the inaugural meta-analysis of MS prevalence and its associated genetic abnormalities.

Our study found a cumulative MS incidence of 17.42%, with a slight male predominance. This rate exceeds that reported in earlier research on newly diagnosed cases (10). Our finding aligns with the observations made by Fianchi et al. (51), who reported a decline in MS incidence from 11% to 7% when assessed at the time of AML diagnosis. In terms of molecular genetics, our meta-analysis identified *FLT3*-ITD mutations as the most frequently linked to MS, with a pooled prevalence of 17.5%. These figures align closely with prior research: Ansari-Lari et al. (21) found these mutations in 15% of MS cases. Pemmaraju et al. (16) and Shallis et al. (3) also reported the *FLT3* mutation as the predominant mutation. The second most common mutations were *MLL* and *NPM1*, with a pooled prevalence of approximately 17%, as highlighted by the studies of Chang et al. (52) and Eckardt et al. (10). According to Chang et al. (1), the *MLL* gene mutation, especially the classic 11q23 abnormality but excluding t(9;11), has been associated with extramedullary involvement and remains a poor prognostic factor. Additionally, Falini et al. (3) recorded *NPM1* mutations in 14% of 181 MS samples. In a larger cohort of 89 AML patients, Ovcharenko et al. (53) observed mutated *NPM1* in 13 out of 15 MS patients. Another key finding from our study is that *DNMT3A* and *TET2* mutations emerged as the third and fourth most common genetic aberrations, respectively.

AML with t(8;21)(q22;q22.1); *RUNX1::RUNX1T1* represents a distinct subtype of AML. Classified as a core-binding factor leukemia, this form of AML is characterized by frequent genetic recurrence and generally has a favorable prognosis (54). Saia et al. delved into the *RUNX1::RUNX1T1* rearrangement in mouse models, shedding light on its frequent association with extramedullary disease (55). Consistent with our data, the predominant fusion gene detected in MS was *RUNX1::RUNX1T1*, demonstrating a cumulative prevalence of 28.10%. This finding accords with the work of Hu et al. and Velagala et al., and it emphasizes the significance of the *RUNX1::RUNX1T1* fusion gene (5, 7). In contrast, certain studies have underscored the sporadic nature of the *RUNX1::RUNX1T1* fusion, noting its presence in just 2% to 3% of MS cases (56). It is noteworthy that significant statistical heterogeneity was observed throughout all fusion gene analyses, likely amplified by varying baseline characteristics among the considered studies.

Geographical variations also influence the genetic profiling in MS. The *NPM1* mutation was prominently observed among patients in Western regions, whereas *KRAS* predominated in those from Eastern countries. Additionally, *FLT3*-ITD was identified as a commonly occurring mutation in both populations. Core-binding fusion genes were frequently observed in AML with MS across continents, albeit with differences in specific fusion genes (*CBFB::MYH11* in the Western population and *RUNX1::RUNX1T1* in the Eastern population).

When comparing genetic abnormalities between non-MS and MS cases, our analysis revealed no significant differences in the prevalence of the *RUNX1::RUNX1T1* and *CBFB::MYH11* fusion genes. However, we found that the *NRAS* mutation was significantly associated with the MS group. Our findings suggest that the *CEBPA* mutation might confer a protective effect against MS, supported by an OR of 0.51 (95% CI 0.32 to 0.81).

This study examined the prevalence of mutations and fusion genes in AML with multiple MS. The findings revealed variations in the incidence rates of certain mutations between the MS group and AML patients without MS. Furthermore, age and geographical disparities emerged as significant factors influencing the genetic profiling in MS cases. Consequently, a mutational workup should be conducted in all newly diagnosed AML patients with MS, as the results offer valuable insights for risk stratification, guiding treatment decisions, and potentially introducing novel therapeutic options targeting specific mutations.

However, this study has several limitations. First, it drew upon published data, potentially introducing publication bias since studies with positive or novel outcomes are more likely to be published than those with negative or neutral findings. Second, numerous analyses indicated elevated I^2^ values, which signify substantial heterogeneity among studies. This heterogeneity might have undermined the reliability of our combined results. Third, some included studies lacked details of patients’ baseline characteristics, and there was evident statistical inconsistency in the genetic testing methods used. Additionally, the source of genetic data, whether derived from bone marrow or blood samples, was not always clearly specified, potentially leading to inaccurate representation of genetic variations. Fourth, the limited number of studies comparing gene mutations between MS and non-MS may have resulted in insufficient statistical power to establish significant differences. Fifth, the included studies utilized a range of techniques, such as conventional cytogenetics, Fluorescence *In Situ* Hybridization, polymerase chain reaction, and NGS, each with varying sensitivities in detecting mutations. This diversity in methods may have influenced the accurate assessment of mutation prevalence. Lastly, the potential relationship between MS and gene mutations is an intriguing area of study; however, we were unable to perform such an analysis in this study due to insufficient data.

## Conclusion

This study underscores the importance of three gene mutations—*FLT3*-ITD, *MLL*, and *NPM1*—which were commonly observed in cases of MS. The fusion gene *RUNX1::RUNX1T1* emerged as the principal genetic fusion associated with MS. Intriguingly, although the *CEBPA* mutation appeared to confer some protection against MS, the presence of the *NRAS* mutation was associated with an elevated risk of developing MS. In essence, this meta-analysis substantially augments our comprehension of the genetic mutation characteristics of MS.

## Data availability statement

The original contributions presented in the study are included in the article/**Supplementary Material**. Further inquiries can be directed to the corresponding author.

## Author contributions

SU: Conceptualization, Data curation, Investigation, Methodology, Resources, Validation, Visualization, Writing – original draft. ST: Conceptualization, Data curation, Investigation, Methodology, Resources, Visualization, Writing – original draft, Writing – review & editing. KK: Conceptualization, Data curation, Investigation, Methodology, Writing – original draft. TR: Conceptualization, Data curation, Investigation, Methodology, Writing – original draft. NL: Conceptualization, Formal analysis, Investigation, Writing – review & editing. SP: Conceptualization, Data curation, Investigation, Methodology, Writing – original draft. TK: Conceptualization, Data curation, Investigation, Methodology, Writing – original draft. WO: Conceptualization, Data curation, Formal analysis, Funding acquisition, Investigation, Methodology, Project administration, Resources, Software, Supervision, Validation, Visualization, Writing – original draft, Writing – review & editing.
