# Supplementary material for: Genetic alterations in myeloid sarcoma among acute myeloid leukemia patients: insights from 37 cohort studies and a meta-analysis
Source: Front Oncol. 2024 Mar 1;14:1325431. doi: 10.3389/fonc.2024.1325431 (PMC10940330; doi:10.3389/fonc.2024.1325431)
Supplement: Supplementary file 1 [file DataSheet_1.docx]

**Supplementary Data 1. Search Strategies**

**MEDLINE**

1. acute myeloid leukemia.mp. or exp Leukemia, Myeloid, Acute/

2. myeloid sarcoma.mp. or exp Sarcoma, Myeloid/

3. exp Leukemic Infiltration/ or leukemic cutis.mp.

4. extramedullary.mp.

5. 2 or 3 or 4

6. 1 and 5

**EMBASE**

1. ‘granulocytic sarcoma’/exp OR ‘granulocytic sarcoma’

2. leukemic AND (‘cutis’ /exp OR cutis)

3. extramedullary

4. ‘acute myeloid leukemia’/exp OR ‘acute myeloid leukemia’

5. #1 or #2 or #3

6. #4 and #5

**SCOPUS**

1. (myeloid AND sarcoma) OR (leukemic AND cutis) OR (extramedullary)
2. (acute AND myeloid AND leukemia)
3. #1 and #2
