# Supplementary material for: Genetic alterations in myeloid sarcoma among acute myeloid leukemia patients: insights from 37 cohort studies and a meta-analysis
Source: Front Oncol. 2024 Mar 1;14:1325431. doi: 10.3389/fonc.2024.1325431 (PMC10940330; doi:10.3389/fonc.2024.1325431)
Supplement: Supplementary file 3 [file DataSheet_3.docx]

**Supplementary Data 3.** The pooled prevalence of mutations and fusion genes in AML patients with myeloid sarcoma categorized by age group

| **Molecular mutations** | **Number of included studies** | **Age <40 years**  **% (95% CI), I^2^** | **Age ≥40 years**  **% (95% CI), I^2^** |
| --- | --- | --- | --- |
| *NPM1* | 3 | 25.40 (-6.90-57.80), 47.43 | 19.80 (4.20-35.40),  0.00 |
| Signal transduction pathway | | | |
| *FLT3*-ITD | 2 | 16.60 (-12.50-45.80), 10.63 | 9.40 (-5.30-24.20), 19.24 |
| *NRAS* | 3 | 21.60 (-5.10-48.40), 0.00 | 11.10 (-2.20-24.40), 19.26 |
| *KIT* | 3 | 10.10 (-3.40-23.60), 0.00 | 18.10 (-0.70-36.80), 0.00 (N = 2) |
| *FLT3*-TKD | 3 | 12.10 (-3.30-27.50), 0.00 | 7.00 (-5.00-19.00),  0.00 (N = 2) |
| *JAK2* | 3 | 12.90 (-6.80-32.60), 0.00 | 13.20 (0.90-25.40),  0.00 |
| *KRAS* | 2 | 50.00 (10.00-90.00), 0.00 | 5.50 (-5.00-16.00),  0.00 |
| Myeloid transcription factor | | | |
| *BCORL1* | 2 | 10.3 (-6.10-26.60),  0.00 | 16.70 (-13.20-46.50), NA (N = 1) |
| *CEBPA* | 2 | 35.7 (-9.70-81.10),  0.00 | 6.60 (-6.00-19.20),  0.00 |
| Tumor suppressor gene | | | |
| *TP53* | 3 | 18.30 (-8.40-45.00), 0.00 | 7.90 (-2.90-18.60),  0.00 |
| Epigenetic modifier | | | |
| *TET2* | 2 | 19.40 (-15.10-53.90), 0.00 | 15.30 (-4.30-34.90), 0.00 |
| *IDH2* | 3 | 13.50 (-7.50-34.40), 0.00 | 20.50 (4.00-37.00),  0.00 |
| *ASXL1* | 4 | 11.70 (-2.60-26.10), 0.00 | 13.30 (-0.90-27.50), 0.00 |
| Spliceosome gene | | | |
| *SRSF2* | 2 | 19.40 (-15.10-53.90), 0.00 | 21.90 (-0.30-44.20), 0.00 |
| Cohesion gene | | | |
| *STAG2* | 3 | 19.40 (-15.10-53.90), 0.00 | 19.70 (-0.40-39.80), 0.00 |
| Fusion gene | | | |
| *RUNX1::RUNX1T1* | 2 | 7.50 (-4.20-19.30), 14.21 | 2.90 (-2.70-8.60),  NA (N = 1) |

N: Number of included studies
