# Supplementary material for: Genetic alterations in myeloid sarcoma among acute myeloid leukemia patients: insights from 37 cohort studies and a meta-analysis
Source: Front Oncol. 2024 Mar 1;14:1325431. doi: 10.3389/fonc.2024.1325431 (PMC10940330; doi:10.3389/fonc.2024.1325431)
Supplement: Supplementary file 4 [file DataSheet_4.docx]

**Supplementary Data 4.** AML Patients with myeloid sarcoma and recurrent genetic abnormalities: (A) pooled prevalence; and (B) risk assessment

**
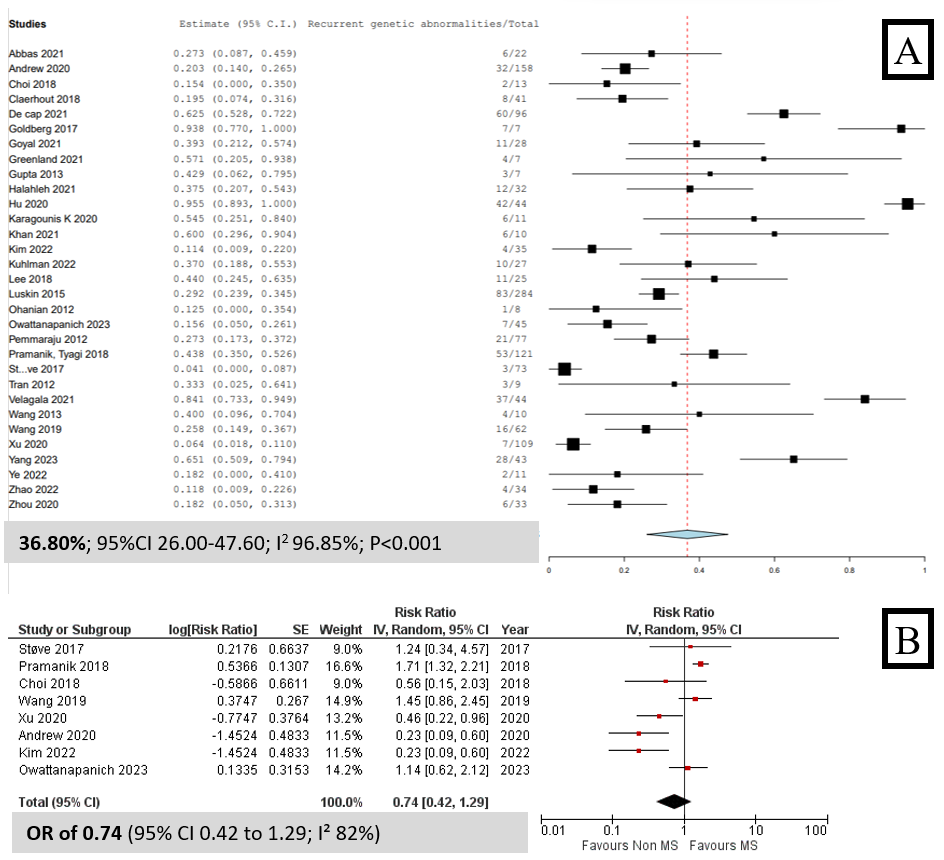
**
