# Supplementary material for: Genetic alterations in myeloid sarcoma among acute myeloid leukemia patients: insights from 37 cohort studies and a meta-analysis
Source: Front Oncol. 2024 Mar 1;14:1325431. doi: 10.3389/fonc.2024.1325431 (PMC10940330; doi:10.3389/fonc.2024.1325431)
Supplement: Supplementary file 5 [file DataSheet_5.docx]

**Supplementary Data 5.** Pooled prevalence of gene mutations in AML patients with myeloid sarcoma in Western countries

| **Molecular mutations** | **Number of included studies** | **% (95% CI)** | **I^2^** |
| --- | --- | --- | --- |
| *NPM1* | 12 | 27.5 (17.8, 37.3) | 87.18 |
| Signal transduction pathway | | | |
| *FLT3*-ITD | 8 | 20.5 (13.9, 27.1) | 77.52 |
| *NRAS* | 9 | 10.9 (7.0, 14.8) | 40.87 |
| *KIT* | 3 | 1.4 (-2.2, 5.1) | 83.43 |
| *FLT3*-TKD | 5 | 9.3 (4.0, 14.5) | 57.70 |
| *PTPN11* | 2 | 0.4 (-0.3, 1.1) | 0.00 |
| *JAK2* | 6 | 4.8 (1.9, 7.7) | 17.31 |
| *KRAS* | 2 | 4.0 (2.1, 5.8) | 0.00 |
| *SH2B3* | ND | - | - |
| *CBL* | ND | - | - |
| *BRAF* | 2 | 1.0 (-4.9, 7.0) | 9.84 |
| Myeloid transcription factor | | | |
| *BCORL1* | ND | - | - |
| *RUNX1* | 4 | 7.7 (0.6, 14.9) | 75.43 |
| *ETV6* | 2 | 3.5 (-0.3, 7.4) | 60.09 |
| *CEBPA* | 5 | 10.7 (3.0, 18.4) | 0.00 |
| Tumor suppressor gene | | | |
| *WT1* | 3 | 5.4 (-5.6, 16.4) | 44.28 |
| *TP53* | 4 | 10.5 (4.2, 16.7) | 11.81 |
| *NF1* | 2 | 3.6 (-2.1, 9.3) | 0.00 |
| *PHF6* | ND | - | - |
| Epigenetic modifier | | | |
| *MLL* | ND | - | - |
| *DNMT3A* | 4 | 12.1 (-2.3, 26.5) | 90.18 |
| *TET2* | 6 | 15.8 (12.4, 19.2) | 0.00 |
| *KMT2A* | 2 | 19.9 (-15.0, 54.9) | 72.89 |
| *IDH2* | 5 | 12.9 (9.7, 16.0) | 0.00 |
| *ASXL1* | 6 | 7.9 (4.4, 11.4) | 22.21 |
| *IDH1* | 4 | 9.8 (6.9, 12.6) | 0.00 |
| *EZH2* | ND | - | - |
| *SETD2* | 2 | 3.6 (-2.1, 9.3) | 0.00 |
| *SETBP1* | ND | - | - |
| Spliceosome gene | | | |
| *SRSF2* | 2 | 10.6 (4.7, 16.6) | 0.00 |
| *U2AF1* | 2 | 6.7 (-1.4, 14.7) | 16.6 |
| *SF3B1* | ND | - | - |
| Cohesion gene | | | |
| *STAG2* | 3 | 12.8 (0.7, 24.8) | 0.00 |
| Fusion gene |  |  |  |
| *BCR::ABL1* | ND | - | - |
| *CBFB::MYH11* | 6 | 20.4 (7.2, 33.5) | 88.31 |
| *RUNX1::RUNX1T1* | 4 | 6.3 (3.2, 9.3) | 0.00 |
| *KMT2A::MLLT3* | 2 | 19.2 (-14.6, 53.0) | 79.63 |
