# Supplementary material for: Genetic alterations in myeloid sarcoma among acute myeloid leukemia patients: insights from 37 cohort studies and a meta-analysis
Source: Front Oncol. 2024 Mar 1;14:1325431. doi: 10.3389/fonc.2024.1325431 (PMC10940330; doi:10.3389/fonc.2024.1325431)
Supplement: Supplementary file 6 [file DataSheet_6.docx]

**Supplementary Data 6.** Pooled prevalence of gene mutations in AML patients with myeloid sarcoma in Eastern countries

| **Molecular mutations** | **Number of included studies** | **% (95% CI)** | **I^2^** |
| --- | --- | --- | --- |
| *NPM1* | 10 | 7.2 (2.9, 11.5) | 75.82 |
| Signal transduction pathway | | | |
| *FLT3*-ITD | 10 | 18.1 (5.9, 27.2) | 82.73 |
| *NRAS* | 3 | 20.1 (8.9, 31.3) | 7.93 |
| *KIT* | 6 | 15.2 (9.4, 21.0) | 0.00 |
| *FLT3*-TKD | 3 | 4.0 (-1.2, 9.3) | 0.00 |
| *PTPN11* | ND | - | - |
| *JAK2* | 2 | 10.4 (-1.2, 22.1) | 0.00 |
| *KRAS* | 4 | 3.2 (-0.2, 6.6) | 0.00 |
| *SH2B3* | ND | - | - |
| *CBL* | 3 | 2.7 (-0.7, 6.1) | 0.00 |
| *BRAF* | ND | - | - |
| Myeloid transcription factor | | | |
| *BCORL1* | ND | - | - |
| *RUNX1* | 3 | 6.8 (1.5, 12.1) | 0.00 |
| *ETV6* | 2 | 10.6 (2.1, 19.1) | 5.92 |
| *CEBPA* | 4 | 3.1 (-0.3, 6.5) | 23.76 |
| Tumor suppressor gene | | | |
| *WT1* | ND | - | - |
| *TP53* | 4 | 2.7 (-0.2, 5.6) | 0.00 |
| *NF1* | ND | - | - |
| *PHF6* | ND | - | - |
| Epigenetic modifier | | | |
| *MLL* | 2 | 6.6 (-5.5, 18.7) | 77.24 |
| *DNMT3A* | 4 | 10.6 (1.7, 19.7) | 56.48 |
| *TET2* | 3 | 13.5 (6.3, 20.6) | 0.00 |
| *KMT2A* | ND | - | - |
| *IDH2* | 4 | 3.6 (-0.6, 7.9) | 17.01 |
| *ASXL1* | 4 | 5.7 (1.1, 10.3) | 0.00 |
| *IDH1* | 4 | 2.3 (-0.4, 4.9) | 0.00 |
| *EZH2* | 2 | 9.4 (1.6, 17.2) | 0.00 |
| *SETD2* | ND | - | - |
| *SETBP1* | 2 | 3.3 (-2.0, 8.5) | 0.00 |
| Spliceosome gene | | | |
| *SRSF2* | 3 | 5.1 (-0.0, 10.3) | 13.23 |
| *U2AF1* | 2 | 3.3 (-2.0, 8.5) | 0.00 |
| *SF3B1* | ND | - | - |
| Cohesion gene | | | |
| *STAG2* | ND | - | - |
| Fusion gene |  |  |  |
| *BCR::ABL1* | 3 | 1.6 (-1.1, 4.3) | 40.05 |
| *CBFB::MYH11* | 7 | 6.4 (1.9, 10.9) | 76.00 |
| *RUNX1::RUNX1T1* | 9 | 21.5 (10.1, 32.9) | 95.69 |
| *KMT2A::MLLT3* | ND | - | - |
